# Public health implications of overscreening for carotid artery stenosis, prediabetes, and thyroid cancer

**DOI:** 10.1186/s40985-018-0095-6

**Published:** 2018-07-02

**Authors:** Bich-May Nguyen, Kenneth W. Lin, Ranit Mishori

**Affiliations:** 1 Memorial Family Medicine Residency Program, 14023 Southwest Freeway, Sugar Land, TX 77478 USA; 2 Department of Family Medicine, Georgetown University School of Medicine, 4000 Reservoir Road, N.W, Washington, D.C., 20007 USA

**Keywords:** Screening, Overtesting, Overscreening, Overdiagnosis, Overtreatment, Carotid artery stenosis, Prediabetes, Thyroid cancer

## Abstract

**Background:**

Overscreening occurs when people without symptoms undergo tests for diseases and the results will not improve their health. In this commentary, we examine three examples of how campaigns to screen and treat specific vascular, metabolic, and oncologic diseases in asymptomatic individuals have produced substantial overdiagnosis and may well have contributed to more harm than good. These conditions were chosen because they may not be as well known as other cases such as screening for breast or prostate cancer.

**Main text:**

Screening for carotid artery stenosis can be a lucrative business using portable equipment and mobile vans. While this fatty buildup of plaque in the arteries of the neck is one risk factor for ischemic stroke, current evidence does not suggest that performing carotid dopplers to screen for CAS reduces the incidence of stroke or provide long-term benefits. After a positive screening, the follow-up procedures can lead to heart attacks, bleeding, strokes, and even death. Similarly, many organizations have launched campaigns for “prediabetes awareness.” Screening for prediabetes with a blood sugar test does not decrease mortality or cardiovascular events. Identifying people with prediabetes could lead to psychological stress and starting medication that may have significant side effects. Finally, palpating people’s necks or examining them with ultrasounds for thyroid cancer is common in many countries but ineffective in reducing mortality. Deadly forms of thyroid cancer are rare, and the overall 5-year survival rate is excellent. Interventions from treatment for more prevalent, less aggressive forms of thyroid cancer can lead to surgical complications, radiation side effects, or require lifelong thyroid replacement therapy.

**Conclusions:**

Screening for carotid artery stenosis, prediabetes, and thyroid cancer in an asymptomatic population can result in unnecessary, harmful, and costly care. Systemic challenges to lowering overscreening include lack of clinician awareness, examination of conflicts of interests, perverse financial incentives, and communication with the general public.

## Background

The overuse of medical services, defined as services that are more likely to harm than benefit patients, has been recognized increasingly as a pervasive worldwide problem [1]. One important driver for overuse is routine testing for disease in asymptomatic persons, also known as screening. Although screening tests for selected conditions have reduced disease-specific mortality, many exams may lead to significant harms including bleeding, infection, injuries, anxiety, and possibly offsetting increased mortality from additional diagnostic procedures and treatments [2].

To achieve the full benefits of an effective population-based screening, the test must be used in an appropriate population, within the appropriate age range, and at appropriate intervals. According to Ebell and Herzstein, overscreening occurs when “tests are performed in asymptomatic persons when there is no evidence that the screening will improve patient outcomes” [3]. Overscreening is distinct from overdiagnosis, which occurs when “a disease is diagnosed and treated, even though that disease never would have harmed the patient if left untreated” [4]. Overdiagnosis can occur even in patients who are appropriate candidates for a screening test. For example, the National Lung Screening Trial demonstrated that annual low-dose CT scans reduced lung cancer and all-cause mortality in heavy smokers [5]. Nonetheless, nearly one in five lung cancers detected in the trial were overdiagnosed, and for every lung cancer death prevented, 1.4 lung cancers were overdiagnosed [5]. Table 1 summarizes common examples of overscreening [6].

**Table 1:** Examples of common screening tests that are sometimes used outside of evidence-based recommendations [6]

Area	Screening tests often ordered unnecessarily	Common occurrences against (US) recommended clinical indications
Infectious diseases	Hepatitis C antibody	Screening outside of age group recommendations; those not at risk
	Human immunodeficiency virus (HIV) antibody	Screening outside of age group recommendations; those not at risk
	Herpes antibody	Screening in general
	Gonorrhea/chlamydia	Screening outside age group recommendations; those not at risk
	Human papilloma virus (HPV)	Screening outside age group recommendations; those not at risk; disregard intervals
Cardiovascular diseases	Electrocardiogram (EKG)	Screening asymptomatic
	Carotid doppler	Screening asymptomatic
	Ankle brachial index (ABI)	Screening asymptomatic
	Cardiac calcium score	Screening asymptomatic
	Abdominal aortic aneurysm ultrasound	Screening those outside of risk group
	Coronary angiography	Screening asymptomatic groups
	Lipids	Disregard recommended intervals
Endocrine diseases	Hemoglobin A1c	Screening outside of risk group
	Bone mineral density for osteoporosis screening	Screening outside of age group/risk group; disregard recommended intervals
Cancer	Ultrasound kidney cancer	Screening in general
	Thyroid function tests (TFTs) thyroid cancer	Screening in general
	Full body computed tomography (CT)	Screening in general
	Mammogram	Screening outside of age group
	Colorectal cancer screening	Screening outside of age group; disregard recommended intervals
	Prostate cancer	Screening in general; outside of risk group

A previous commentary in *Public Health Reviews* outlined the general causes and consequences of overdiagnosis [7]. In this commentary, we discuss examples of campaigns to screen for three specific vascular, metabolic, and oncologic diseases in asymptomatic individuals and comment on their potential to produce substantial overdiagnosis and most likely cause more harm than good. Screening for carotid artery stenosis, prediabetes, and thyroid cancer was chosen because these illustrations of overscreening may not be as well known as other cases such as screening for breast or prostate cancer.

## Vascular disease: screening for carotid artery stenosis

### Ongoing screen-and-treat campaigns

Calls for “life-saving” health screenings for heart disease and stroke prevention from private businesses—as opposed to doctors and hospitals—are not unique to the United States (US), where companies, such as HealthFair [8] and Lifeline screening [9, 10], bring portable equipment and mobile vans to community centers and charge between 100–400 dollars for screening “packages.” It is becoming a big business in other parts of the world as well, including the United Kingdom and Australia [11–13].

These packages sign people up for tests such as an electrocardiogram or EKG (to look for atrial fibrillation), abdominal aortic ultrasound (to look for aortic aneurysm), and ankle-brachial index test (to look at blood flow in the legs), among others. The organizers do not require a physician’s prescription and perform the tests regardless of age, symptoms, or risk factors. In other words, anyone can have any test without evidence-based decision-making about the appropriateness of some of these tests for screening or diagnostic purposes.

These campaigns and companies also offer carotid artery ultrasound to “detect a blockage or narrowing of your carotid arteries so you can seek the required medical intervention to prevent a stroke from occurring, saving you devastating, irreversible affects to your quality of living” [10]. The carotid artery test—also known as carotid doppler—is painless, easy to do, and non-invasive, using ultrasound waves to create an image of the carotid arteries on each side of the neck. Findings of accumulation of plaque—measured by “percent occlusion”—may lead to recommendations to seek follow-up care with a physician.

### Disease burden and diagnostic criteria

According to the American Heart Association (AHA), stroke ranks fifth among all causes of death in the US and second globally [14, 15]. Each year, approximately 795,000 cases of stroke occur in the US, leading the AHA to offer this staggering statement that “on average, every 40 seconds, someone in the United States has a stroke, and on average, every 4 minutes, someone dies of a stroke” [14].

Many cases of non-fatal stroke, also known as cerebrovascular accidents (CVA), result in debilitating and life-long effects such as paralysis, weakness, cognitive impairment, speech and language problems, visual damage, and eating difficulties, among others.

Carotid artery stenosis (CAS)—a fatty buildup of plaque in the arteries of the neck—is but one risk factor for ischemic stroke. CAS causes approximately 10% of ischemic strokes, and the estimated prevalence in the US populations of asymptomatic CAS is about 1%, making this condition relatively uncommon [14].

CAS may be a consequence of atherosclerosis, hypertension, hyperlipidemia, and diabetes. Other risk factors for CAS include age, smoking, family history, obesity, sleep apnea, and a sedentary lifestyle.

### Lack of evidence that screening improves health outcomes

Most US and international organizations agree that screening low-risk or asymptomatic individuals for carotid artery stenosis using ultrasound is ill advised. The US Preventive Services Task Force (USPSTF) conducted a systematic review and meta-analysis on the benefits and harms of screening for CAS among adults without symptoms. In 2014, it issued a recommendation “against screening for asymptomatic carotid artery stenosis in the general adult population,” assigning it a D grade (“There is moderate or high certainty that the service has no net benefit or that the harms outweigh the benefits”) [16].

The USPSTF evaluators were unable to find any high-quality studies that showed a reduction in instances of stroke for those screened with an ultrasound. Additionally, they found no good evidence that screening conferred any long-term benefits when followed up by invasive interventions such as surgery, angiography or stenting, or intensified medical therapy.

On the contrary, the authors were able to find studies showing that screening resulted in harm—not from the screening test itself, which is painless and harmless, but from the cascade of follow-up procedures. The harms included heart attacks, bleeding, strokes, and even death [17–20].

There is nearly uniform agreement among many US-based and international organizations that CAS screening is not recommended. In the United Kingdom (UK), the National Health Service (NHS) noted, “Prevention methods are currently more effective than screening” [21]. Table 2 is a collection of some organizations’ statements about the utility of CAS.

**Table 2:** International organizations statements about carotid artery stenosis screening

Society for Vascular Surgery	“Carotid artery screening (CAS) is not recommended for asymptomatic patients at this time.” [21, 60]
European Society of Cardiology [61] and the European Stroke Society [62]	“Systematic carotid duplex screening is of limited value.” [61]
The Royal Australian College of General Practitioners (RACGP) in Summary of “screening tests of unproven benefits” [63]	“It is no longer justifiable to screen for the presence of asymptomatic carotid artery stenosis to select patients for carotid procedures. There is no current evidence of patient benefit. However, there is evidence of harms from screening, including significant procedural risk and cost” [63]
Australian Stroke Foundation [64]	“Carotid artery screening for stroke prevention is a highly controversial area and is not endorsed by national or international guidelines.” [64]
The Australian and New Zealand Society for Vascular Surgery [65]	“If you have had no strokes or transient ischemic attacks (TIAs) the need for a scan is more controversial as the risk of stroke is low.” [65]

Other European, Italian, Israeli, and Czech experts and societies have also published similar statements [22–27].

### Overdiagnosis and other screening downsides

Screening adults who have no symptoms for CAS—a condition with a relatively low prevalence—may lead to many false-positive results (in which CAS is incorrectly diagnosed) and/or overdiagnosis (there is some stenosis, but it may not be clinically meaningful). Overdiagnosis of CAS often leads to overtreatment: unnecessary tests and invasive procedures, including a risky surgery, which are costly and potentially harmful. The preponderance of evidence led the US and Canadian Choosing Wisely campaigns [28] to conclude, “There is good evidence that for adult patients with no symptoms of carotid artery stenosis the harms of screening outweigh the benefits.”

## Metabolic disease: screening for prediabetes

### Disease burden and diagnostic criteria

The press release announcing the Centers for Disease Control and Prevention’s 2017 National Diabetes Statistics report was headlined, “More than 100 million Americans have diabetes or prediabetes” [29]. The Centers for Disease Control and Prevention (CDC) report estimated that 30 million Americans have diabetes and another 84 million have prediabetes [30], which most often refers to persons with a hemoglobin A1c level of 5.7 to 6.4% or a fasting plasma glucose level of 100 to 125 mg/dL. “Diabetes prevention” typically refers to helping adults with prediabetes improve their diets, become more physically active, and (if overweight) lose weight to delay or prevent the onset of type 2 diabetes.

### Ongoing screen-and-treat campaigns

In 2015, the CDC and the American Medical Association (AMA) launched the Prevent Diabetes: STAT (Screen, Test, Act Today) campaign [31]. The USPSTF recommended that primary care clinicians screen overweight or obese adults between the ages of 40 and 70 for abnormal blood glucose levels and provide or refer patients at risk for type 2 diabetes to intensive lifestyle counseling [32]. The CDC, AMA, American Diabetes Association (ADA), and the Ad Council are promoting “prediabetes awareness” with public service announcements featuring puppies, hedgehogs, and baby goats that encourage viewers to take a 1-min prediabetes risk test [33]. Starting in April 2018, the Medicare Diabetes Prevention Program (DPP) will pay qualified organizations up to $450 per Medicare beneficiary to provide a lifestyle change intervention modeled after the successful 2002 National Institutes of Health-sponsored DPP trial [34].

### Lack of evidence that screening improves health outcomes

Existing evidence does not show that screening for abnormal blood glucose levels in asymptomatic persons improves patient-oriented health outcomes. In a USPSTF-commissioned systematic review, the largest randomized controlled trial of screening found no mortality benefit after 10 years compared to usual care [35]. A later population-based controlled trial of screening for type 2 diabetes and cardiovascular risk factors found no differences between the intervention and control groups in mortality or cardiovascular events [36]. Finally, a systematic review of screening tests and treatments for prediabetes confirmed the short-term effectiveness of lifestyle interventions in reducing the incidence of diabetes but found that “fasting glucose is specific but not sensitive and HbA1c is neither sensitive nor specific” for identifying persons with prediabetes [37].

### Overdiagnosis and other screening downsides

The ADA has lowered their diagnostic criteria for diabetes and prediabetes multiple times since 1997, despite scant proof that lifestyle change or medications improve health outcomes for additional diagnosed patients [38–40], and no other accepted rationale for modifying disease definitions [41]. The online risk test promoted by the ADA, CDC, and AMA would classify 60% of individuals 40 years or older and 80% of individuals 60 years or older as being at “high risk for prediabetes” [42]. A test with such low specificity, followed by an inaccurate diagnostic test for a pre-condition that has relatively weak predictive value for developing diabetes, is a recipe for a lot of overdiagnosis in primary care.

Consider the numbers. If one third of US adults (33 out of 100) has prediabetes [30], but studies indicate that fewer than one third of these persons (say 10 out of 100) will progress to diabetes within 10 years [43], the screening campaign would potentially give 23 out of every 100 adults a prediabetes diagnosis without measurable health benefits. On the other hand, this diagnosis could harm by causing psychological stress or generating prescriptions for medications such as metformin, which may have a variety of side effects.

## Oncologic disease: screening for thyroid cancer

### Disease burden and diagnostic criteria

Welch and Black proposed characteristics for overdiagnosis specific to cancer: (1) a condition that commonly has a subclinical or asymptomatic disease reservoir, (2) activities (such as mammograms and blood tests) enabling detection of cases from the disease reservoir, and (3) a population-level mismatch in rates of change between incidence and mortality [44]. The diseases most commonly discussed in relation to this framework are breast and prostate cancers. However, other malignancies such as thyroid cancer fulfill these characteristics as well. Screening for thyroid cancer in asymptomatic individuals involves neck palpation by a health professional looking for masses or imaging with ultrasound. Increased use of imaging technologies also leads to finding incidental thyroid nodules that trigger evaluations for thyroid carcinoma, including blood tests and biopsies.

There are four main types of thyroid cancer. Two types have excellent prognosis, and the other two are more aggressive [45]. Risk factors include certain genetic conditions, a family history, exposure to radiation, and lack of iodine in the diet [46]. Yet, thyroid cancer is relatively rare, representing 3.4% of all cancers and showing an incidence rate of 14.2 per 100,000 men and women per year [47]. The number of deaths was 0.5 per 100,000 men and women per year, and the overall 5-year survival rate is 98.2% [47].

Despite these excellent statistics, people without symptoms are excessively and needlessly screened for thyroid cancer. Overscreening is common in South Korea and the US and possibly in other countries such as Australia, Canada, China, Croatia, the Czech Republic, France, Israel, and Italy [48, 49].

### Lack of evidence that screening improves health outcomes

While the incidence of thyroid cancers in some countries has increased, experts believe it is disproportionately due to increased detection of the less-aggressive forms. The growth in recognition has not led to a reduction in mortality [50]. In fact, in patients with one or more thyroid masses larger than 5 mm, malignancy was noted only in 1.6% [51]. Given that most masses or nodules turn out to be benign, and that most of detected cases of thyroid cancers will turn out to be one of the less aggressive forms, there appears to be little benefit for routine screening of thyroid cancer, which may be overdiagnosing the disease [52].

### Overdiagnosis and other screening downsides

While there is limited research on the direct harms of thyroid cancer screening from direct physical examination to imaging with an ultrasound, there are known harms from the likely follow-up, which may include biopsies, thyroid surgery, and radioactive iodine therapy. Patients diagnosed with thyroid cancer may undergo total thyroidectomy, neck lymph node dissection, and radiotherapy. As with any invasive interventions such as neck biopsies and surgery, patients risk bleeding, infection, and airway obstruction caused by bleeding. Partial or total removal of the thyroid may cause additional complications such as hypoparathyroidism and vocal cord paralysis or require lifelong thyroid replacement therapy [48]. Radioactive iodine therapy has its own short- and long-term effects, including disruption of saliva production, and hormonal effects that can affect fertility. By nature of its toxic radioactive effects, this therapy can also increase the chances of developing other types of cancer [53, 54].

In addition to risks from potential evaluation and treatment, the cost can be high. The initial and continuing surveillance of the less aggressive thyroid cancers was estimated to cost the US over $1.6 billion in 2013 for patients diagnosed after 1985 [55]. Based on current trends, this analysis anticipates the cost of care in the US in 2030 will exceed $3.5 billion [55].

There are limited recommendations from international organizations about thyroid cancer screening for general populations. The National Evidence-based Healthcare Collaborating Agency and Korean Thyroid Association concluded in 2013 that the current evidence was insufficient to determine whether thyroid ultrasound screening decreased morbidity or mortality from thyroid cancer [56]. In 1996 and again in 2017, the USPSTF recommended against routine thyroid cancer screening in asymptomatic adults [57].

## Conclusions

This commentary furthers the knowledge described by Bulliard and Chiolero [7] with exploration of three specific examples illustrating common instances of overscreening. Screening for a variety of conditions—endocrine, cardiovascular, and malignant, among others—in a general, asymptomatic population can result in unnecessary, harmful, and costly care. In order to change the pervasive culture of overscreening and overdiagnosis, we must overcome several systemic challenges. Those include lack of clinician awareness of evidence-based recommendations, high-quality systematic reviews that examine authors’ financial conflicts of interests, and the presence of financial incentives to carry out procedures and unnecessary imaging studies.

Efforts are underway, however, to resolve some of these factors, chief among them the international Choosing Wisely campaign—now in the US, Canada, UK, and Italy, among other countries—in which professional medical societies rely on the evidence to make practice recommendations to their members about unnecessary care [58].

One aspect that has proven a bit tougher to combat on the road to practicing high-value screening and care is public demand for some of these tests in the false hopes that “catching a disease early” will result in avoiding serious disease and early death. It is essential to communicate with the public about the harms from overdiagnosis without adding confusion or distrust [59]. Clinicians must work with researchers, public health experts, the media, and other stakeholders to ensure that evidence-based health messages do not fall on deaf ears.

## Abbreviations

ABI: Ankle brachial index
ADA: American Diabetes Association
AHA: American Heart Association
AMA: American Medical Association
CAS: Carotid artery stenosis
CDC: Centers for Disease Control and Prevention
CT: Computed tomography
CVA: Cerebrovascular accident
DPP: Diabetes Prevention Program
EKG: Electrocardiogram
HIV: Human immunodeficiency virus
HPV: Human papilloma virus
NHS: National Health Service
RACGP: Royal Australian College of General Practitioners
STAT: Screen, Test, Act Today campaign
TFTs: Thyroid function tests
TIA: Transient ischemic attack
UK: United Kingdom
US: United States
USPSTF: United States Preventive Services Task Force
